# Nasopharyngeal carcinoma-associated inflammatory cytokines: ongoing biomarkers

**DOI:** 10.3389/fimmu.2024.1448012

**Published:** 2024-10-17

**Authors:** Chuwen Liang, Jun Kan, Jingli Wang, Wei Lu, Xiaoyan Mo, Bei Zhang

**Affiliations:** ^1^ TCM&VIP Inpatient Department, Sun Yat-sen University Cancer Center, Guangzhou, China; ^2^ State Key Laboratory of Oncology in South China, Guangdong Key Laboratory of Nasopharyngeal Carcinoma Diagnosis and Therapy, Guangdong Provincial Clinical Research Center for Cancer, Sun Yat-sen University Cancer Center, Guangzhou, China

**Keywords:** nasopharyngeal carcinoma, inflammation, cytokines, predictive factors, prognostic factors

## Abstract

Nasopharyngeal carcinoma (NPC) is a neoplasm related to inflammation; the expression of cytokines, such as CCL3, CCL4, CCL20, IL-1α, IL-1β, IL-6, IL-8, and IL-10, among others, is presumed to be associated with NPC occurrence and development. Therefore, the circulating levels of these cytokines may be potential biomarkers for assessing tumor aggressiveness, exploring cellular interactions, and monitoring tumor therapeutic responses. Numerous scholars have comprehensively explored the putative mechanisms through which these inflammatory factors affect NPC progression and therapeutic responses. Moreover, investigations have focused on elucidating the correlation between the systemic levels of these cytokines and the incidence and prognosis of NPC. This comprehensive review aims to delineate the advancements in research concerning the relationship between inflammatory factors and NPC while considering their prospective roles as novel prognostic and predictive biomarkers in the context of NPC.

## 1 Introduction

Nasopharyngeal carcinoma is a malignant tumor originating from the nasopharyngeal epithelium and is primarily localized within the pharyngeal recess (fossa of Rosenmüller) (1–3). According to the World Health Organization (WHO) classification, NPC can be divided into three pathological subtypes: keratinizing squamous, nonkeratinizing squamous and basaloid squamous tumors. It has significant regional distribution differences worldwide and is prevalent mainly in Southeast Asia, North Africa and southern China (4). Within the above endemic areas, the predominant subtype is nonkeratinizing squamous carcinoma, which is closely related to Epstein–Barr virus (EBV) infection (2, 3, 5).

Although early-stage NPC patients have significant enhancements in local control and overall survival (OS) rates, owing to the insidious nature of NPC progression and the anatomical intricacies of the nasopharynx, a substantial proportion of patients in endemic regions are diagnosed at advanced stages (3, 6–9). Moreover, patients the same tumor−node−metastasis (TNM) stages usually present with disparate clinical outcomes, indicating that the TNM staging system is inadequate for prognostication or therapeutic stratification (8, 10). Therefore, there is an urgent need to explore novel tumor markers with high specificity and sensitivity for large-scale population screening and to identify biomarkers that can complement the existing clinical staging system to improve the accuracy of prognosis prediction to facilitate the application of more effective and suitable treatment methods. Many studies have confirmed that cytokines, which serve as important regulatory mediators of the inflammatory response by acting locally in a paracrine or autocrine manner, constitute complex networks to mediate the interaction between the tumor and the host; they not only affect the tumor microenvironment (TME), but also cause systemic reactions by traversing into the systemic circulation, which highlights the roles of cytokines as pivotal regulatory factors in the pathophysiology of cancer (11–20). In breast cancer, lung cancer, colorectal cancer, prostate cancer, etc., they may function as predictive and prognostic factors (21–27).

There are also numerous relevant reports on NPC. By comprehensively analyzing the correlation between the expression levels of different inflammatory cytokines and pathogenesis, as well as the correlation between the expression levels and key prognostic indicators, such as incidence, survival duration, and recurrence rates, in patients with NPC, we hope to reveal the clinical value of serum cytokine levels as potential predictive and prognostic biomarkers. The in-depth discussion of this review provides a theoretical basis for a better understanding of the inflammatory mechanisms of NPC and the development of individualized precision treatments for patients in the future.

## 2 Cancer and inflammation

In the preceding century, scholars reported significant infiltration of inflammatory cells within tumor tissues, sparking early recognition of a potential relationship between inflammation and tumorigenesis, which led to the hypothesis that “inflammation may be one of the causes of tumor formation” and gradually led to the conclusion that the relationship between inflammation and tumors can be divided into two pathways: the intrinsic pathway and the extrinsic pathway (12, 14, 20, 28). In the former pathway, tumorigenesis is initiated via genetic events; thus, cells that arise in this way release inflammatory mediators, thereby instigating an inflammatory microenvironment within tumors to promote angiogenesis and tumor growth (12, 14, 29). The second pathway is thought to constitute the classic pattern by which NPC arises; in this pathway, inflammatory processes precede tumorigenesis. In this case, a sustained inflammatory microenvironment is established as a consequence of persistent infections or chronic inflammatory diseases, increasing susceptibility to cancer or facilitating tumorigenesis and metastasis (12, 14, 20).

**Figure 1:**
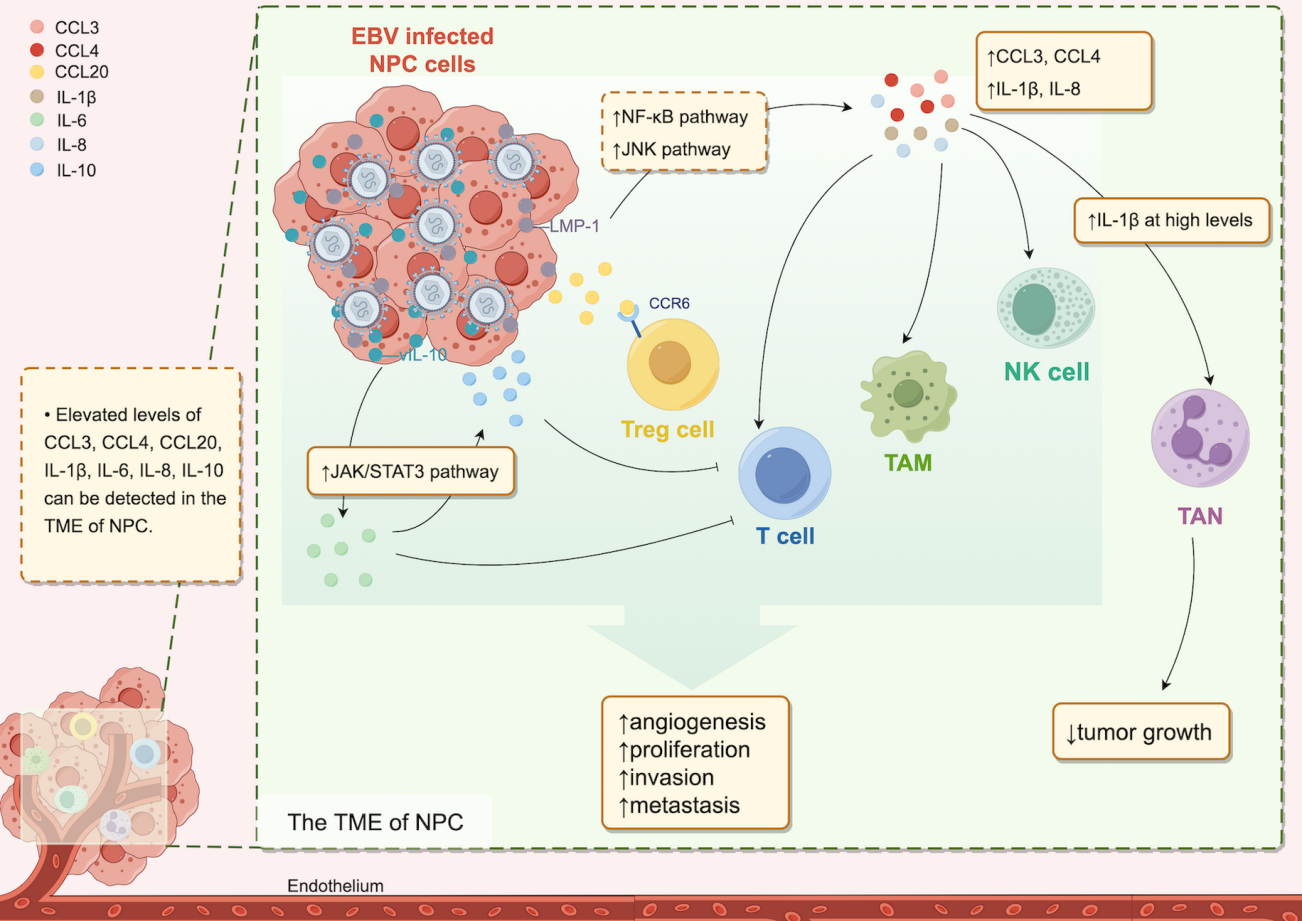
Cytokines in the TME of NPC. In the TME of NPC, increased levels of various cytokines are detectable. The EBV-infected cell product, LMP-1, upregulates CCL3, CCL4, IL-1β, and IL-8 expression via the NF-kB and JNK pathways, and vIL-10 induces IL-6 expression through the JAK2/STAT3 pathway, leading to increased IL-10 downstream. Cytokines present at elevated levels in the TME attract diverse immune cells, resulting in a protumor effect. However, high IL-1β levels are associated with antitumor effects.

These two pathways ultimately merge via a shared mechanism, establishing a persistent inflammatory state within the milieu of malignant cells and their surrounding microenvironment, which affects tumorigenesis. Therefore, certain scholars have characterized inflammation as the “incubator” of the TME (20). In this inflammatory microenvironment, many inflammatory cytokines, such as CCL3, CCL4, CCL20, IL-1α, IL-1β, IL-6, IL-8, and IL-10, are expressed and operate through diverse cellular signaling pathways, playing important roles in inflammatory cell recruitment and activation, malignant transformation of tissue cells, tumor angiogenesis, and tumor cell immune evasion, as well as invasion and metastasis (13, 19, 30–40) (**Figure 1**).

## 3 Inflammatory factors associated with NPC

NPC is an example of a malignancy closely associated with inflammation. In addition to its strong correlation with EBV infection, the significant infiltration of inflammatory cells in tumor tissues and EBV-related cytokine expression further substantiate this notion (13, 41). The substantial infiltration of nonmalignant leukocytes in the TME is among the reasons for the abnormal expression of inflammatory factors and local suppressed immune surveillance (42, 43). Consequently, tumor-derived cytokines in the peripheral blood of NPC patients may represent potential biomarkers for assessing tumor invasiveness, indirectly exploring cell interactions, and monitoring therapeutic efficacy. Analyzing blood cytokine concentrations may be a simple and cost-effective means of monitoring tumor occurrence and progression (13, 44, 45).

While inflammatory factors typically function locally through paracrine or autocrine mechanisms, various inflammatory cytokines, such as CCL3, CCL4, CCL20, IL-1α, IL-1β, IL-6, IL-8, and IL-10, have been identified at aberrant levels in the bloodstream of individuals with nasopharyngeal carcinoma and are correlated with the incidence and prognosis of NPC.

### 3.1 The CC family of chemokines

The CC family of chemokines is one of the chemokine families, alongside the CXC, C, and CX3C chemokine families. Chemokines are involved in the development, differentiation and migration of leukocytes. They are also integral in processes such as angiogenesis, wound healing, inflammatory disorders, and the pathogenesis of malignancies such as NPC, liver cancer, and colorectal cancer. Although some chemokines exhibit antitumor effects by inducing the infiltration of cytotoxic T lymphocytes into tumors and killing tumor cells, certain chemokines inevitably have protumorigenic effects related to their ability to recruit immunosuppressive cells (46–49) (**Table 1**).

**Table 1:** The CC family of chemokines and their roles in nasopharyngeal carcinoma.

Cytokine	Produced by	Function in NPC	Mechanism involved	Possible clinical value	Ref.
CCL3	Tumor cells	A potent attractant for CD3^+^CD8^+^ T cells	Upregulated through activates NF-κB and JNK pathways by LMP-1	–	(52)
		–	–	A marker to predict the risk of NPC	(54)
		–	–	Improve diagnostic efficiency	(55)
CCL4	Tumor cells	A potent attractant for CD3^+^CD4^+^ T cells	Upregulated through activation of NF-κB and JNK pathways by LMP-1	–	(52)
	Tumor cells and vascular endothelial cells	Selective recruitment of CCR5^+^ T cells	–	–	(53)
	Tumor cells	–	–	A marker to predict the risk of NPC	(54)
CCL20	Tumor cells	Mediates the migration and invasion of NPC cells *in vitro*	–	Serves as a useful predictor and prognosticator for NPC	(58)
	NPC tissue	–	–	Improves screening effi-ciency; positively cor-related with EBV DNA load and TNM stage	(6)
				An independent prognostic factor for OS, DMFS, LRFS of NPC patients	(8)

NPC, nasopharyngeal carcinoma; CCL, C-C motif chemokine ligand; NF-κB, nuclear factor-kappa B; JNK, c-Jun N-terminal kinase; LMP-1, latent membrane protein-1; CCR5, chemokine receptor 5; EBV, Epstein–Barr virus; TNM, tumor node metastasis; OS, overall survival; DMFS, distant metastasis-free survival; LRFS, local recurrence-free survival.

#### 3.1.1 CCL3, CCL4

CCL3 and CCL4, formerly known as macrophage inflammatory protein (MIP)-1α and MIP-1β, are highly related members of the CC chemokine subfamily and are synthesized by diverse cells, including neutrophils, lymphocytes, macrophages, and epithelial cells (46, 47). They both play important roles in recruiting leukocytes to sites of infection, delivering interferons to mediate protective responses against viral infections, and inducing antitumor responses (30, 50). An abnormal concentration of CCL4 is detectable in human head and neck squamous cell carcinoma (HNSCC), but the association between its level and the prognosis of HNSCC remains controversial (44, 51).

In the context of NPC, CCL3 or CCL4 can be expressed by malignant cells and tumor vascular endothelial cells (30, 52–55). CCL3 can recruit inflammatory cells actively expressing the chemokine receptors CCR1 and CCR5, whereas CCL4 selectively induces the accumulation of inflammatory cells that overexpress CCR5 within the TME (46, 47, 53). Both chemokines can recruit T-cell subsets, such as CD8^+^ and CD4^+^ cells, as well as monocyte/macrophage and NK cells; however, CCL3 and CCL4 exhibit differential preferences in recruiting T-cell subsets, with CCL3 showing a predilection for CD8^+^ T cells, whereas CCL4 tends to recruit CD4^+^ T cells (52, 53). Furthermore, Lai et al. reported that in EBV-associated NPC, increased expression of EBV latent membrane protein (LMP)-1 can cause continuous activation of the NF-κB and JNK signaling pathways, thereby inducing the upregulation of CCL3 and CCL4 expression in tumor cells, leading to increased T-cell infiltration and a poor prognosis in NPC patients (52).

Yang et al. conducted a two−stage epidemiologic study elucidating a potential association between diminished preclinical levels of CCL3 and CCL4 in southern China populations and a subsequent heightened risk of NPC, irrespective of EBV infection (54). In contrast, Xue et al. reported the upregulation of CCL3 expression in NPC malignant cells, concomitant with a notable increase in the serum CCL3 concentration among patients compared with that in their healthy counterparts (55). They further highlighted that a three-factor assessment of macrophage migration inhibitory factor (MIF), EBV capsid antigen (VCA)-IgA, and CCL3 greatly increased the positive predictive value (PPV) and sensitivity of NPC screening, and this approach distinguished NPC patients from healthy people more accurately than traditional approaches (55).

Yang et al. reported that the expression levels of CCL4 were significantly greater than those of CCL3. Since CCL4 mainly recruits CD4^+^ T cells, CCL4 upregulation may lead to a greater proportion of infiltrating regulatory T (Treg) cells in the tumor and enhance local immunosuppression (52–54). Thus, CCL4 upregulation supports NPC cell evasion of the antitumor immune response and consequently increases the risk of developing NPC. Conversely, Xue et al. did not detect a statistically significant difference in CCL4 concentration in the cohort (55). This may be one of the reasons why the two studies led to opposite conclusions. Therefore, to evaluate the predictive and prognostic utility of these serum biomarkers, further exploration of the expression differences and influencing factors of CCL3 and CCL4 before and after treatment in larger cohorts is necessary.

#### 3.1.2 CCL20

CCL20, also known as MIP-3α, is constitutively expressed in the lymph nodes, liver, lung, etc. (56). However, in the TME of several malignancies, including hepatocellular carcinoma, colorectal carcinoma, and breast carcinoma, the upregulation of CCL20 expression is detectable (56). This upregulation facilitates oncogenic effects through the CCL20/CCR6 signaling axis, which directly facilitates cancer cell migration and proliferation and indirectly promotes cancer progression by recruiting Treg cells expressing high levels of CCR6, thereby fostering the formation of an immunosuppressive TME (48, 56, 57).

Chang et al. reported elevated expression of CCL20 in NPC cells and confirmed the ability of CCL20 to promote tumor metastasis *in vitro (* 6). CCL20 levels are positively correlated with tumor T stage (6, 8, 53). In addition, positive correlations between pretreatment serum CCL20 concentration and overall tumor stage and between posttreatment serum CCL20 concentration and indicators such as recurrence and metastasis rates were found in a prospective cohort (6). Moreover, subsequent investigations revealed a significant decrease in posttreatment serum MIP-3α levels compared with baseline levels (6, 8). Importantly, NPC patients who experienced recurrence or distant metastasis posttreatment presented markedly higher serum MIP-3α levels than long-term survivors (6, 8). Consistent with this study, other studies have shown that adding CCL20 to existing NPC screening assays can increase the efficiency of initial screening and that high levels of serum CCL20 may indicate a poor NPC prognosis (8, 58).

Hence, utilizing CCL20 as a diagnostic biomarker and prognostic indicator holds significant promise; however, large-scale validation studies are warranted for further confirmation.

### 3.2 Interleukin families

The interleukin family constitutes a critical class of inflammatory cytokines that play pivotal roles in the intricate network of intercellular interactions, together with other types of cytokines, which are implicated in tumorigenesis and tumor progression (18).

LMP-1, in addition to eliciting the upregulation of the abovementioned chemokines via the NF-κB and JNK pathways to advance EBV-associated NPC, also induces the expression of diverse interleukins, such as IL-1β, IL-8 and their analogs, through the activation of the NF-κB and STAT3 signaling pathways, which can lead to leukocytes that provide immunosuppressive effects infiltrating, and pathological progression of tumors (31, 32, 43, 59). IL-6 has emerged as a significant factor in NPC pathogenesis. It can induce the activation of STAT3, leading to the activation of JAK/STAT3 downstream signaling and ultimately promote the proliferation, invasion and metastasis of NPC cells (31). Therefore, many studies have explored the potential of IL-1α, IL-1β, IL-6, IL-8, and IL-10 as new diagnostic or prognostic markers for NPC (**Tables 2A**, **B**).

**Table 2A:** Interleukin family members and their roles in nasopharyngeal carcinoma.

Cytokine	Produced by	Functions in NPC	Mechanism involved	Ref.
IL-1α/IL-1β	LMP1-expressing epithelial cells	Contribute to lymphocyte infiltration and/or tumor growth during NPC development	Their production is mediated by LMP-1 through NF-κB pathway and could be enhanced by TNF-α	(33)
	EBV-positive epithelial cells and infiltrating CD4^+^ T cells		–	(62)
IL-1β	Tumor cells that express LMP-1	Low levels of tumor-derived IL-1β can facilitate tumor growth, but high levels can inhibit tumor growth and local relapse by recruiting neutrophils	LMP-1 can induce pro-IL-1β expression through NF-κB and MAPK pathways, then inflammasomes cleave pro-IL-1β to IL-1β	(64)
IL-6	NPC tissue	Enhances tumor cell proliferation, migration and invasion	The upstream activator of JAK2/STAT3 pathway	(34)
	All tested NPC cell lines	Promotes the migration and invasion of NPC cell lines and upregulates the expression of MMP-2 and MMP-9	Establishes a positive feedback loop of IL-6 induction, promotes STAT3 phosphorylation, and stabilizes LMP-1 expression	(70)
IL-8	Tumor cells	Promotes angiogenesis in NPC	Expression can be induced by MIF	(80)
			Expression can be induced by LMP-1	(76)
			LMP-1 induces the expression of IL-8 mainly through NF-κB pathway, and AP-1 pathway only plays a partial role	(35)
	NPC cell line, NPC-KT			(36)
	NPC cell line, CNE-2 cells		Expression can induce by MIF by activating the NF-κB and AP-1	(79)
	High-metastasis NPC cells	Promotes the migration, invasion, and metastasis abilities of NPC cells	Activates AKT signaling and the induction of EMT	(40)
IL-10	NPC tissue	Inhibits tumor-specific cytotoxic T cells	Expression can be induced by LMP-1	(86)
	EBV-infected tumor cells			(38)
		An immunosuppressive growth factor for T cells and cytokine production	Downregulates signal trans-duction activated by B7 receptors, thereby suppressing the function of cytotoxic T cells	(87)
	Tumor cells and infiltrating MNCs	Enhances the survival and the growth of host B cells and suppresses the immunologic function	–	(88)

NPC, nasopharyngeal carcinoma; IL, interleukin; LMP-1, latent membrane protein-1; NF-κB, nuclear factor-kappa B; TNF-α, tumor necrosis factor-alpha; EBV, Epstein–Barr virus; MAPK, mitogen-activated protein kinase; JAK2, Janus kinase 2; STAT3, signal transducer and activator of transcription 3; MMP, matrix metalloproteinase; MIF, migration inhibitory factor; AP-1, activator protein-1; EMT, epithelial–mesenchymal transition; MNCs, mononuclear cells.

**Table 2B:** Interleukin family members and their roles in nasopharyngeal carcinoma.

Cytokine	Possible clinical value	Ref.
IL-1β	High levels of tumor-derived IL-1β are associated with better survival in NPC patients after treatment	(64)
	An important factor of NPC progression	(65)
	Higher posttreatment serum levels of IL-1β indicate a good therapeutic response and a better survival rate.	(67)
IL-6	Upregulation of IL-6 is related to the degree of lymph node involvement and TNM stage and correlated with decreased survival time in NPC patients	(34)
	Positively associated with the severity of NPC and EBV DNA loads	(58)
	A promising target for preventing and inhibiting NPC metastasis	(70)
	An important factor in NPC progression	(65)
	High pretreatment levels indicate high mortality rate	(67)
	An independent indicator of poor OS and DMFS for nonmetastatic NPC patients undergoing radical radiotherapy, and concentration changes are valuable for prediction during follow-up	(71)
	Serves as a marker for disease progression and a poorer prognosis in NPC patients	(73)
	An independent indicator of OS, DFS, DMFS and lung-MFS; CI-model, which considers both sIL6 level and TNM stage, has the highest prediction efficiency for the outcome of NPC patients	(74)
	A promising marker for presence and therapeutic assessment of NPC	(75)
IL-8	Not a discussion of serum IL-8 level; the expression levels in the primary tumor could be an independent prognostic factor for OS, DFS, and DMFS of patients with NPC	(40)
	Higher in advanced overall stages and poorer OS	(58)
	No discussion on serum IL-8 levels; significantly correlated with advanced clinical stage and decreased survival rate but not an independent prognostic factor	(80)
	Significant correlated with advanced N classification and clinical stage but not an independent prognostic marker for NPC	(81)
	A promising marker for the presence and progression of NPC; positively correlated with EBV DNA load	(82)
	An independent indicator of a poor prognosis and associated with an increased risk of relapse/death	(84)
IL-10	No discussion on serum IL-10 levels; the local expression of IL-10 is negatively correlated with N stage	(86)
	Elevated in patients with undifferentiated carcinoma and late stage	(87)
	No discussion on serum IL-10 levels; IL-10 expression *in situ* was a significant independent prognostic indicator of OS	(88)

IL, interleukin; NPC, nasopharyngeal carcinoma; JAK2, Janus kinase 2; STAT3, signal transducer and activator of transcription 3; TNM, tumor node metastasis; EBV, Epstein–Barr virus; OS, overall survival; DMFS, distant metastasis-free survival; DFS, disease-free survival; lung-MFS, lung metastasis-free survival; sIL6, serum interleukin 6.

#### 3.2.1 IL-1 family

The IL-1 family encompasses interleukins such as IL-1α, IL-1β, IL-33, etc., each of which play multifaceted roles in the regulation of carcinogenesis and tumor progression (18, 60). Tumor type, stage of progression, and TME features are important factors that determine the effect of IL-1 on cancer; for example, in the context of malignancy, IL-1α and IL-1β predominantly facilitate tumor growth; however, during early malignant transformation, they may exhibit antitumor properties (61).

As early as the 1980s and 1990s, researchers sequentially observed enriched expression of IL-1α and IL-1β within NPC tissues, particularly in EBV-positive epithelial cells as well as infiltrating CD4^+^ T cells, and proposed the hypothesis that they may serve as factors driving lymphocytic infiltration and/or tumor progression during the development of NPC (62, 63). Experimental evidence conducted *in vitro* has corroborated this notion, further suggesting that the upregulation of IL-1 in NPC tissues results from the induction of LMP-1 through the NF-κB signaling pathway, which can be positively modulated by tumor necrosis factor (TNF)-α (33). In addition to regulating lymphocytic infiltration, increased levels of tumor-derived IL-1β (with a threshold of 64.2 pg/ml in murine models) have been shown to recruit a plethora of tumor-associated neutrophils (TANs) to significantly suppress tumor growth (64). Consequently, patients experience prolonged local recurrence-free survival (LRFS) and disease-free survival (DFS) (64). Nonetheless, Chen et al. also suggested that tumor-derived IL-1β may exert a growth-promoting effect when it is present at low levels (64). In other clinical cohorts, elevated serum concentrations of IL-1α/IL-1β have been detected in pretreatment samples from NPC patients compared with healthy control samples, and Al-Kholy et al. reported that clinical stage was significantly negatively correlated with the serum IL-1β level; however, the trend in the variation in posttreatment IL-1β levels was controversial among these studies (65–67).

To date, comprehensive investigations into the diagnostic efficacy and prognostic significance of IL-1α and IL-1β are lacking. Given their dual roles in carcinogenic processes, further study is needed to determine the suitability of these interleukins as biomarkers.

#### 3.2.2 IL-6

Unlike IL-1, which has dual effects, IL-6 is a prototypical protumorigenic cytokine that promotes chronic inflammation while supporting tumor angiogenesis and inhibiting Th1 cell-mediated antitumor immunity (68, 69). Exosomes derived from NPC cells can significantly induce IL-6 production from macrophages, then, IL-6 significantly increased the malignant behaviors of NPC cells by regulating various oncogenic processes mediated by STAT3 or upregulating the expression of matrix metalloproteinase (MMP)-2 and MMP-9, thereby promoting NPC cells migration and invasion (19, 34, 70–72).

Studies have consistently indicated a correlation between elevated levels of IL-6 in the blood and a poor NPC prognosis (34, 58, 67, 71, 73, 74). Tan et al. reported a significant reduction in IL-6 levels following NPC treatment, suggesting a potential indirect reflection of improved inflammatory status within the TME and indicating treatment efficacy in patients (75). Other investigations have corroborated this observation (65, 67, 71). Zhuang et al. confirmed that the expression levels of the IL-6 and JAK2/STAT3 signaling pathway components in NPC were associated with TNM stage, lymph node metastasis and a decreased survival rate (34). Inhibition of the expression of these factors can suppress the invasion, proliferation and migration of NPC cells (34). These findings substantiate the viewpoint posited by Tan et al.

However, the relationship between IL-6 levels and advanced TNM stage at the initial diagnosis of NPC remains unclear (34, 58, 73–75). The divergent findings regarding this association underscore the limitation of relying solely on TNM staging for comprehensive prognostication in patients. Ke et al., through robust statistical analysis of a large cohort, established a prognostic model termed the CI model, which integrates baseline serum IL-6 levels (sIL6) with NPC TNM staging (74). This model exhibits superior predictive accuracy compared with TNM staging alone, offering enhanced prognosis assessment of OS, DFS, and other pertinent outcomes in NPC patients (74).

Combined with the above findings, findings in additional validation studies on the correlation between changes in the IL-6 concentration during treatment and the above prognostic indicators in a large cohort, as well as finding on the accuracy of this model, may position IL-6 as the most rapidly deployable prognostic marker among inflammatory mediators in clinical practice.

#### 3.2.3 IL-8

IL-8, commonly referred to as CXCL8, can exert angiogenic effects, which are crucial for tumor sustenance by ensuring that sufficient oxygen and nutrients are available for growth, thereby potentially underpinning tumorigenesis and metastasis (18, 76, 77). MIF has been identified as a lymphokine capable of activating the immune system (78). In NPC, MIF expression levels are positively correlated with those of IL-8, and MIF is posited as a putative upstream modulator that promotes IL-8 induction and consequently stimulates tumoral neovascularization (79, 80). Another inducer of IL-8 expression in NPC pertains to EBV infection—LMP-1 can increase the transcriptional activity of IL-8 via the NF-κB pathway and the c-jun kinase pathway (35, 36). In addition to its angiogenic role, *in vitro* studies revealed that IL-8 can also promote the motility of NPC cells and lead tumor dissemination through activating the AKT signaling pathway and inducing epithelial–mesenchymal transition (36, 40).

The functional characteristics of IL-8 support that its expression closely related to the prognosis of NPC. Elevated levels of IL-8 demonstrate a pronounced increase in tandem with the advancement of both N classification and TNM stage (58, 81). Investigations by Li et al. have revealed that increased IL-8 expression within primary NPC tissues is an independent prognostic factor that significantly impacts patient outcomes, including OS, DFS, and DMFS (40). Consistent with this result, other studies have also reported a correlation between the baseline serum IL-8 concentration and poor prognosis in NPC patients, suggesting that IL-8 has high potential value as a prognostic biomarker in the context of NPC management (58, 81–84).

#### 3.2.4 IL-10

IL-10 has been defined as a suppressive immune regulatory factor for long (85). It is synthesized by diverse immunocytes and attenuates immune reactivity while suppressing the release of stimulatory cytokines, thereby inhibiting the proliferation and effector functions of T cells and leading to the formation of an immunosuppressive TME (38, 39, 85). In EBV-associated NPC, virally infected cells express a protein closely resembling human IL-10, denoted as viral IL-10 (vIL-10), which can upregulate the expression of IL-6 through the JAK2/STAT3 signaling pathway (37, 38). Concomitantly, IL-6 increases STAT3 phosphorylation, thereby inducing IL-10 expression (31, 39). This process may augment the oncogenic potential of IL-6, promoting the proliferation and metastasis of NPC cells while concurrently supporting the immunosuppressive TME via IL-10.

The formation of an immunosuppressive TME promotes the immune escape of NPC cells, leading to increased tumor invasiveness (38, 86, 87). Fujieda et al. reported that the expression of IL-10 *in situ* was closely related to patients’ clinical outcomes, with IL-10-positive patients exhibiting markedly shorter survival times than their IL-10-negative counterparts (88). Moreover, a greater proportion of IL-10-positive cells is correlated with increased mortality risk (88). In addition, Budiani et al. reported elevated circulating IL-10 levels in advanced-stage NPC patients (87). In contrast, Tan et al. failed to discern statistically significant changes in serum IL-10 concentrations among healthy controls and NPC patients pre/posttreatment, suggesting the localized expression of IL-10, primarily within the TME, without substantial systemic secretion (75).

While IL-10 plays a pivotal role in tumor advancement, existing studies do not provide adequate evidence to substantiate an association between IL-10 concentrations in the peripheral blood of NPC patients and the intratumoral expression of IL-10. Whether the serum IL-10 level can be detected by serological tests to determine prognosis needs further study.

## 4 Conclusion and perspective

In contrast to the diagnostic gold standard of tumor-histological biopsy, screening of tumor biomarkers in serum is less invasive and potentially more cost-effective and widely applicable approach (44).

Currently, screening for newly diagnosed NPC predominantly involves serological assays targeting anti-EBV IgA antibodies, specifically early antigen (EA)-IgA, VCA-IgA, and EBV nuclear antigen 1 (EBNA1)-IgA (2, 3, 89). However, approximately 95% of the world’s population is continuously infected with asymptomatic EBV, and the associated high false-positive rate in antibody testing limits its suitability for screening in endemic regions (54, 90–92). Furthermore, although circulating EBV DNA has been demonstrated to be a promising indicator for NPC screening and prognosis evaluation, transient elevations in EBV DNA may occur in healthy individuals. In addition, the demands of sophisticated procedures and specialized equipment and the lack of a standardized detection protocol of quantitative analysis of EBV DNA contribute to significant interlaboratory variability (2, 3, 6, 7, 9, 93). Consequently, reliance solely on EBV-related biomarkers is insufficient. Scholars are seeking more cost-effective and reliable biomarkers for NPC screening, therapeutic efficacy prediction, and prognosis assessment.

There is increasing interest in the indispensable role of inflammation in the evolution of NPC. Researchers are presently delving into the interrelationship between the expression profiles of inflammatory mediators within afflicted individuals and pertinent prognostic indices, hoping to reveal the clinical value of serum inflammatory factor levels as predictive and prognostic biomarkers. In the study of Chang et al., the levels of IL-6, IL-8, CCL4 and CCL20 were significantly different between patients with NPC and patients with chronic rhinosinusitis, suggesting that although inflammatory diseases in adjacent organs may cause an increase in cytokine markers, the abnormal elevation of these markers can still indicate the development of tumors (58). However, prior to a possible clinical application, several difficulties have to be overcome. For example, our knowledge regarding the functional characteristics of these cytokines in TME remains limited. The relationship between elevated levels of these factors *in situ* and elevated blood concentrations needs to be further explored. Besides, the future clinical cohorts must take more other processes different from malignancy that might affect cytokine levels into consideration.

This article provides a comprehensive summary of recent research on eight cytokines, namely, CCL3, CCL4, CCL20, IL-1α, IL-1β, IL-6, IL-8, and IL-10. The review is structured around two key facets: elucidating their procarcinogenic or anticarcinogenic mechanisms and summarizing advancements in research regarding their expression levels and their correlation with tumorigenesis and cancer prognosis. We hope to provide a direction for further research to determine the relationships between specific inflammatory factors and the pathogenesis, progression and therapeutic responses of NPC.
